# Evaluation of Depth of Anesthesia Sleep Quality in Swine Undergoing Hernia Repair: Effects of Romifidine/Ketamine-Diazepam Protocols with and Without Tramadol and the Potential Role of Serotonin as a Biomarker

**DOI:** 10.3390/vetsci12080722

**Published:** 2025-07-31

**Authors:** Fabio Bruno, Fabio Leonardi, Filippo Spadola, Giuseppe Bruschetta, Patrizia Licata, Veronica Cristina Neve, Giovanna Lucrezia Costa

**Affiliations:** 1 Department of Veterinary Sciences, University of Messina, 98168 Messina, Italy; fabio.bruno@unime.it (F.B.); fspadola@unime.it (F.S.); giuseppe.bruschetta@unime.it (G.B.); patrizia.licata@unime.it (P.L.); veronicacristina.neve@unime.it (V.C.N.); 2 Department of Veterinary Science, University of Parma, 43126 Parma, Italy; fabio.leonardi@unipr.it

**Keywords:** tramadol, swine, depth anesthesia, quality sleep, serotonin, biomarker, multimodal analgesia

## Abstract

Sedation and anesthesia are essential for ensuring animal welfare during surgical procedures such as hernia repair in swine. However, the number of sedative and anesthetic agents officially approved for livestock use remained limited. This study evaluated the sedative efficacy and serotonergic effects of a romifidine/ketamine/diazepam protocol, with and without the addition of tramadol, in swine undergoing umbilical hernia repair. Sixty-six crossbred Large White swine were randomly allocated to three groups: LL (lidocaine 4 mg/kg by infiltration), LT (lidocaine 2 mg/kg by infiltration + tramadol 2 mg/kg intraperitoneally), and TT (lidocaine2 mg/kg by infiltration + tramadol 4 mg/kg intraperitoneally). The physiological parameters heart rate, arterial pressure, oxygen saturation, rectal body temperature, and respiratory rate were assessed. The depth of intraoperative anesthesia and postoperative sedation was assessed using an ordinal scoring system (0–3). Plasma serotonin (5-HT) concentration was measured at baseline and 24 h post-surgery. Physiological parameters remained within species-specific reference ranges throughout the procedure. Anesthesia depth scores significantly decreased over time in all groups (*p* ≤ 0.001), with the tramadol-treated groups (LT and TT) showing more prolonged deeper anesthesia. Postoperative sedation was significantly higher in the TT group (*p* ≤ 0.001). Serotonin concentration decreased in LL, increased in LT, and remained stable in TT. These findings suggest that tramadol may enhance sedation and recovery, potentially through serotonergic modulation. Moreover, serotonin could serve as a physiological marker warranting further investigation in future studies of anesthetic protocols in veterinary medicine.

## Simple Summary

This study evaluated the effects of supplementing a standard anesthetic protocol (comprising romifidine, ketamine, and diazepam) with tramadol, a widely used analgesic. In addition to its analgesic effects mediated via opioid receptor activation, tramadol also modulates central serotonin levels, which are closely associated with sleep regulation and postoperative recovery. A total of 66 swine received romifidine, ketamine, and diazepam and were randomly assigned to one of three treatment groups. The control group received only lidocaine as a local anesthetic; the second group received lidocaine plus a low dose of tramadol; and the third group received lidocaine plus a high dose of tramadol. The depth of anesthesia, physiological parameters, and serotonin concentrations were assessed intraoperatively and postoperatively. Swine treated with tramadol exhibited deeper sedation, more stable physiological parameters, improved recovery profiles, and stable or elevated serotonin levels compared to the control group. These findings suggest that tramadol may enhance both the depth of anesthesia and the quality of postoperative recovery, potentially through serotonin-mediated neurophysiological mechanisms. The results support the integration of tramadol into multimodal sedation protocols and highlight serotonin as a promising biomarker for assessing sedation quality in veterinary practice.

## 1. Introduction

The use of sedatives and anesthetics in veterinary medicine is essential to ensure the welfare of animals undergoing surgical procedures. In swine, during major surgeries such as hernia repair, the choice of sedative and anesthetic agents can have a significant impact on both the quality of anesthesia and postoperative recovery. However, only a limited number of sedatives and anesthetics are approved for use in livestock worldwide [1]. Romifidine is an alpha-2 adrenergic agonist that provides both sedation and analgesia, making it a widely used agent in veterinary practice. Its use in swine has been shown to produce effective sedation and analgesic with minimal cardiovascular side effects [2]. The combination of ketamine, a dissociative anesthetic, and diazepam, a benzodiazepine, is also commonly employed due to its rapid onset and potent sedative properties. The synergy between these two agents may enhance both the depth of sedation and the quality of analgesia during surgery [3].

However, these drugs are not registered for use in swine in Italy and are therefore administered under the “cascade” provision, allowing off-label use under veterinary discretion. Tramadol is a centrally acting analgesic widely used in both human and veterinary medicine, characterized by a dual mechanism of action: activation of μ-opioid receptors and inhibition of serotonin and norepinephrine reuptake [2,4]. In humans, its primary analgesic effect is mediated via μ-opioid receptor agonism [5]. In contrast, studies in dogs have demonstrated minimal affinity for μ-receptors, with the principal mechanism of action attributed to monoaminergic modulation [6].

In swine, available data are limited; however, some studies suggest that tramadol’s analgesic effects in this species may be mediated predominantly through serotonergic and noradrenergic pathways rather than strong opioid receptor activation. Vullo et al. reported measurable antinociceptive effects following tramadol administration in pigs, without the occurrence of typical opioid-related adverse effects, thus supporting a non-opioid mechanism of action [7]. These findings underscore the need for further pharmacodynamic and pharmacokinetic studies to better understand tramadol’s mode of action in swine and to optimize its use in veterinary analgesic protocols.

When tramadol is combined with sedatives and anesthetics, it may enhance their effectiveness, supporting the development of balanced anesthesia protocols [8]. The inclusion of tramadol in such protocols may not only improve analgesia but also positively influence sleep quality during and after surgery. Given the critical role of sleep in postoperative recovery, examining its relationship with serotonin levels may offer valuable insights for optimizing anesthetic strategies [8]. Serotonin, or 5-hydroxytryptamine (5-HT), is a biogenic amine neurotransmitter that plays a crucial role in a range of physiological processes within both the peripheral and central nervous systems [9]. In the peripheral nervous system (PNS), serotonin modulates gastrointestinal motility, vasoconstriction, blood pressure regulation, inflammation, and bone metabolism [10]. In the central nervous system (CNS), it is involved in mood regulation, sleep, appetite, thermoregulation, anxiety, and memory [10,11]. Sleep is a complex and multifactorial process that requires the involvement of the neurotransmitter 5-HT with regulatory action on sleep and wakefulness [12]. Serotonin is involved in both arousal and synchronization responses essential for sleep stability during the different phases of sleep and wakefulness, underscoring serotonin’s role as a modulator [13]. Some studies have further delineated the pathways through which serotonin affects the sleep–wake cycle [14]. Arousal is primarily regulated at the thalamic level, whereas the synchronizing effects of sleep originate from structures located in the floor of the fourth ventricle, such as the area postrema and the nucleus of the solitary tract [15]. The nucleus of the solitary tract integrates visceral sensory input and is modulated by serotonergic input from the raphe nuclei, likely via 5-HT1B receptors [16]. This region also contains a high density of opioid receptors, the stimulation of which has been shown to enhance slow-wave sleep (SWS), producing a hypnotic effect [17]. After the discovery of serotonin in the CNS, its role in sleep modulation was widely documented, as well as the injection of low doses of serotonin into the lateral ventricle of cats, which induced biphasic effects, with an initial phase of arousal followed by drowsiness and sleep [9,13,18]. Intravenous serotonin administration similarly induced early arousal followed by prolonged hypersynchrony, particularly in curarized, non-anesthetized cats. In cats that had undergone brainstem transection, serotonin injections caused immediate arousal followed by EEG synchronization [19]. Further investigations into thalamic mechanisms revealed that serotonin injections exert state-dependent effects, suppressing recruiting responses during wakefulness and inducing arousal during sleep, thus highlighting paradoxical biphasic actions [20,21]. Blocking serotonin synthesis with parachlorophenylalanine (pCPA) reduced total sleep time, confirming serotonin’s role in sleep regulation [18,22].

The primary aim of this study was to compare the depth of anesthesia achieved using a romifidine/ketamine/diazepam protocol, with and without the addition of tramadol, in swine undergoing hernia repair.

Particular emphasis was placed on evaluating the effects of tramadol on both anesthesia depth and sleep quality. In addition, serotonin levels were measured as a potential biomarker for the neurophysiological modulation of sleep.

By incorporating current knowledge regarding the biphasic role of the serotonergic system in regulating the sleep–wake cycle, this study aimed to investigate whether tramadol enhances not only sedation but also postoperative recovery outcomes. This potential benefit was hypothesized to occur through improvements in sleep quality.

We hypothesized that the addition of tramadol to a standard romifidine/ketamine-diazepam anesthetic protocol could improve the quality and duration of anesthesia in pigs undergoing hernioplasty, and that this effect was mediated, at least in part, by serotonergic mechanisms. In particular, we postulated that tramadol administration could result in greater stability of intraoperative physiological parameters and a better postoperative recovery profile.

These findings may have important implications for the refinement of balanced anesthesia protocols in veterinary medicine, especially in species such as swine where pharmacological options are limited.

## 2. Materials and Methods

This research received ethical approval from the University of Messina’s Ethics Committee (Protocol No. 027/2018) and was conducted in accordance with Italian regulations (DM 116192), European directives (Official Journal ECL 358/1 dated 18/12/1986), and U.S. laws (Animal Welfare Assurance No. A5594-01, Department of Health and Human Services, Washington, DC, USA), specifically following Legislative Decree No. 193 of 6 April 2006. The appropriate sample size was calculated using G*Power software version 3.1. An a priori power analysis was conducted based on a one-way fixed-effects ANOVA (omnibus test), assuming an effect size (f) of 0.45, an alpha level of 0.05, statistical power (1-β) of 0.80, and three experimental groups (66 subjects). The sample size used in the study was determined with the aim of detecting statistically significant differences among groups with adequate statistical power. The study was conducted during the months of April and May, with average temperatures ranging from 12 °C to 27 °C in April and from 16 °C to 28 °C in May. All pharmacological interventions were documented in the barn’s official treatment log, and written informed consent was obtained from the swine owners. The animals involved were not intended for meat production or commercial use.

### 2.1. Animals

The study included 66 crossbred Large White swine, aged 90 ± 4 days and weighing 45 ± 10 kg. Among them, 11 were males and 55 were females. The inclusion criterion was the presence of an umbilical hernia measuring 3–5 cm in diameter.

The swine were randomly assigned into three groups of 22 animals each (LL, LT, and TT) using a lottery method. Animals with signs of omphalitis were excluded from the study. Animals did not have any other pathological conditions besides the umbilical hernia.

### 2.2. Treatment Administration

One day prior to surgery, the swine was weighed using a Zoopiro scale (Cutro, Italy) Zoopiro scale (Cutro, Italy). After being placed in a holding pen for 30 min to allow for acclimation, the animals underwent a clinical examination. This included the assessment of heart rate, respiratory rate, and rectal temperature; inspection of the oral mucous membranes; auscultation of the heart and lungs; and palpation of the umbilical region. Furthermore, hematological and bio-chemical parameters were evaluated. Day of surgery, to induce anesthesia, a mixture of ketamine at 4 mg/kg (Ketavet Intervet Productions international S.r.l., Latina, Italy)/diazepam at 4 mg/kg (Ziapam Domes Pharma SC, Pont-du-Château, France) and romifidine (80 μg/kg; Sedivet, Boehringer Ingelheim, Rhein, Germany) was administered intramuscularly in a single syringe.

After surgical anesthesia and muscle relaxation were achieved, the swine were positioned on their backs. The umbilical area was disinfected, and a local anesthetic protocol was carried out using tramadol 5% (Altadol, Formevet, Milan, Italy) and lidocaine 2% (Lidocaine, Esteve, Barcelona, Spain).

- LL group: received lidocaine (4 mg/kg) both via infiltration along the surgical site and via intraperitoneal injection.
- LT group: received lidocaine (2 mg/kg) by infiltration and tramadol intraperitoneally (2 mg/kg).
- TT group: received tramadol (4 mg/kg) both by infiltration and intraperitoneally [2].

In the LL and TT groups, the total volume was equally divided between infiltration and intraperitoneal administration. To ensure even tissue distribution, the injection volume for each method was increased to 40 mL using physiological saline. All groups received infiltrative anesthesia across the skin and muscle layers of the umbilical area, while the intraperitoneal injection was delivered directly into the hernia sac [23]. Throughout surgery, swine were administered 0.9% sodium chloride at a rate of 5 mL/kg/h via an infusion set (Medvet Srl, Taranto, Italy) with a flow regulator (5–250 mL/h) [2]. The fluid was delivered through a 14G 5/12 venous catheter inserted into the jugular vein.

### 2.3. Umbilical Hernia Repair

An elliptical incision was made in the skin, and any adhesions between the peritoneum and the dermis were removed. Displaced organs were repositioned, and the hernia ring was isolated and refreshed. The linea alba was sutured using size 2-0 chromic catgut in a horizontal mattress pattern. An autologous flap was formed from the remaining hernia sac. Subcutaneous tissues were closed using size 2-0 chromic catgut with simple interrupted sutures. Excess skin was excised and closed with size 2-0 non-absorbable nylon (DACLON NYLON TR^®^, Vetefarma, Cuneo, Italy) using the same stitching method. Two experienced surgeons performed all procedures together [23].

### 2.4. Monitoring of Physiological Parameters

During anesthesia, the following were continuously monitored using a CAMS 2 multiparameter monitor (Forlì, Italy): heart rate (HR), systolic, diastolic, and mean arterial pressure (SAP, DAP, MAP) via a non-invasive arm cuff (12–19 cm), oxygen saturation (SpO_2_) via a tongue sensor, and rectal body temperature (T°). Respiratory rate (f_R_) was measured by observing thoracic movements.

Measurements were taken at the following time points:

- T0 baseline (awake subject);
- T1: after sternal recumbency;
- T2: after placement in dorsal recumbency;
- T3: at the time of skin incision;
- T4: during muscle incision;
- T5: at hernia sac opening and herniorrhaphy;
- T6: during muscle and subcutaneous tissue closure;
- T7: at the time of skin suturing.

An increase of 20%, associated with surgical stimulation, in heart rate (HR), respiratory frequency (f_R_), and systolic pressure (SAP) was used as the threshold for the administration of rescue analgesia, consisting of a 2 mg/kg bolus of lidocaine, via infiltration along the surgical site or via intraperitoneal injection [2].

The depth of anesthesia was evaluated using a descriptive ordinal scale ranging from 0 to 3. Scoring was performed by three independent observers blinded to the treatment groups. The scale was defined as follows:

- Score 0: the animal was fully alert and responsive;
- Score 1: mild depth of anesthesia characterized by ease of handling with minimal muscle relaxation;
- Score 2: moderate depth of anesthesia, with the animal remaining manageable and exhibiting partial muscle relaxation;
- Score 3: deep level of anesthesia, where the animal was easily handled and displayed marked muscle relaxation.

A score of 1 (mild depth of anesthesia) was used as the threshold for administering a bolus of the anesthetic mixture equal to half the initial loading dose.

Scores were assigned at T0 baseline (awake subject); T1 after sternal recumbency; T2 after to dorsal recumbency; T3 at the time of skin incision; T4 during muscle incision; T5 at hernia sac opening and herniorrhaphy; T6 during muscle and subcutaneous tissue closure; and T7 at the time of skin suturing.

Postoperative sedation was assessed using the modified Richmond Agitation–Sedation Scale (RASS), adapted for use in sedated, mechanically ventilated swine, allowing for standardized evaluation of sedation depth based on behavioral and physiological responses. The measurement scale comprises 10 response options, with scores ranging from +4 (combative) to −5 (unarousable). A score of 0 or higher denotes an unsedated state. Scores of −2 and −3 correspond to light and moderate sedation, respectively, which represent the targeted sedation levels in the swine intensive care unit (ICU) [24]. Post-operative sedation was assessed after the end of surgery and every due 2 h for 4 h. A RASS score of +2 (agitated) was interpreted as indicating pain-related discomfort and flunixin meglumine (3.3 mg/kg; Finadyne, Schering-Plough Animal Health, Oss, The Netherlands) was administered intramuscularly. After post-surgical discomfort monitoring, flunixin meglumine was administered for three days to all swine.

### 2.5. Sample Collection

All biological samples were collected by the same trained operator to ensure consistency. For the quantification of 5-HT, blood samples (5 mL) were drawn from the jugular vein at two time points: baseline (T2) and 24 h post-surgery (T8). The sample was placed into a K3-EDTA-containing tube (Vacuette^®^, Greiner Bio-One, Kremsmünster, Austria). Samples were promptly stored at 4 °C for up to 3 h, followed by centrifugation at 2000× *g* for 10 min at 4 °C to isolate serum and plasma fractions. All laboratory analyses were conducted by investigators blinded to the surgical method applied.

### 2.6. Plasma 5-HT Quantification

Plasma concentrations of 5-HT (ng/mL) were determined in the platelet-poor plasma (PPP) fraction using a commercial enzyme-linked immunosorbent assay (ELISA) kit (Ref. UNFI0018, AssayGenie, Dublin, Ireland & London, UK), following the manufacturer’s instructions. Briefly, 50 μL of either standard or plasma sample was added to each well, followed by 50 μL of biotin-labeled detection anti-body. The plate was incubated for 45 min at 37 °C, then washed three times. Subsequently, 100 μL of streptavidin–HRP conjugate (SABC) working solution was added, and the plate was incubated again for 45 min at 37 °C. After another washing step, 90 μL of TMB substrate solution was introduced to each well, followed by a 45 min incubation at 37 °C [25]. The reaction was stopped by adding 50 μL of stop solution, and absorbance was measured at 450 nm, with 630 nm as the reference wavelength, using a spectrophotometer (A560, Fulltech, Rome, Italy). The assay’s sensitivity was 1 ng/mL, with intra- and inter-assay coefficients of variation reported as 3.8% and 7.7%, respectively.

### 2.7. Statistical Analysis

Data analysis was carried out using SPSS version 27.1 (IBM Corp., Novegro-Tregarezzo, Italy). The Shapiro–Wilk test was employed to evaluate the normality of data distribution. Results were expressed as mean ± standard deviation (SD) or as median with range, depending on data characteristics. Variations in 5-HT across time points and among groups were analyzed using two-way repeated measures ANOVA, followed by Bonferroni post hoc corrections. Differences in intra and post-operative sedation scores over time and among groups were evaluated using the Friedman test. Inter-observer reliability for postoperative pain assessments was determined through Kendall’s coefficient of concordance (W). SPSS performed automatic corrections where applicable, and a *p*-value < 0.05 was considered statistically significant.

## 3. Results

The overall sample consisted of 66 subjects, with an achieved statistical power of 0.8. Inter-observer reliability was excellent (W = 1). The data did not follow a normal distribution. All enrolled swine successfully completed the study.

The recorded physiological parameters were within the normal range for swine, and variables such as heart rate (HR), respiratory rate (f_R_), and systolic arterial pressure (SAP) did not show increases of 20% during surgery. Depth of anesthesia scores significantly increased over time, in comparison to baseline, across all groups (*p* ≤ 0.001) (Table 1).

A comparison of the depth of anesthesia scores revealed no significant differences; however, in the groups that received tramadol, deeper anesthesia was more prolonged.

In the postoperative period, the scores did not show significant changes over time in any of the groups. The comparison between groups regarding postoperative sedation scores revealed significant differences (*p* ≤ 0.001), with the TT group displaying deeper sedation, as indicated by lower scores according to the Richmond Agitation–Sedation Scale (RASS) compared to the LL and LT groups (Table 2).

Plasma serotonin concentrations decreased significantly 24 h after surgery in the LL group (*p* = 0.007). Conversely, a statistically significant increase in plasma serotonin levels was observed in the LT group (*p* ≤ 0.001), while in the TT group, plasma serotonin concentrations remained unchanged (Table 3).

## 4. Discussion

This study investigated the sedative efficacy and serotonergic response associated with romifidine/ketamine/diazepam and local anesthesia with lidocaine or tramadol in swine undergoing hernia repair. Each drug—romifidine, ketamine, diazepam, lidocaine and tramadol—acts on distinct molecular targets and metabolic pathways, and their combined use produces synergistic effects through mechanisms that include modulation of noradrenergic tone, GABAergic inhibition, NMDA receptor antagonism, monoaminergic balance, and sodium channel blockade, collectively contributing to the reduction in sympathetic activation during surgery [2,4]. The findings highlight several aspects of intraoperative sedation quality and postoperative recovery. In particular, the addition of tramadol appeared to enhance the stability of depth of anesthesia, facilitating the management of animals during more stimulating surgical phases. Postoperative recovery assessments suggested that tramadol supplementation may contribute to a smoother and more controlled emergence from anesthesia. This study investigated the depth of anesthesia and serotonergic response associated with two romifidine/ketamine/diazepam-based anesthetic protocols, with and without the addition of lidocaine or tramadol, in swine undergoing hernia repair. The findings highlight several aspects of intraoperative anesthesia quality and postoperative recovery. In particular, the addition of tramadol appeared to enhance the stability of sedation, facilitating the management of animals during more stimulating surgical phases. Postoperative recovery assessments suggested that tramadol supplementation may contribute to a smoother and more controlled emergence from anesthesia. Moreover, the modulation of plasma serotonin levels emerged as a potential biomarker reflecting neurophysiological processes related to anesthesia. Changes in serotonin concentrations indicate an active involvement of this neurotransmitter in mediating sedative responses and maintaining neuronal synchronization during pharmacologically induced unconsciousness. The results support the hypothesis that the addition of tramadol to the romifidine/ketamine/diazepam protocol, with or without lidocaine, may enhance anesthetic efficacy and improve recovery quality. Furthermore, the findings provide novel insights into the potential role of plasma serotonin as an objective biomarker of sedation depth in experimental animal models.

All physiological parameters measured during the study, including heart rate (HR), respiratory rate (f_R_), and systolic arterial pressure (SAP), remained within species-specific reference intervals throughout the surgical procedures. Similar findings were observed in a previous study in which, during hernia repair in swine, a general anesthesia protocol was employed that included induction with romifidine, tiletamine/zolazepam, and local anesthesia with tramadol and lidocaine administered both along the incision lines and into the hernia sac. Notably, no increases exceeding 20% were observed in these variables during surgery, indicating a stable anesthetic depth and absence of noxious-induced sympathetic activation across all treatment groups [2,3].

Depth of anesthesia scores showed a statistically significant reduction over time in all experimental groups (*p* ≤ 0.001), indicating a progressive decline in depth of anesthesia level consistent with the expected pharmacokinetic profiles, of the drugs used [2,3]. Although no statistically significant differences in depth of anesthesia scores were found among the groups during the intraoperative period, the animals receiving tramadol exhibited more prolonged and deeper anesthesia qualitatively. This observation may reflect the additive or synergistic sedative and analgesic properties of tramadol when used in association with alpha-2 agonists, dissociative anesthetics, and benzodiazepines [2].

Postoperatively, sedation scores did not significantly change over time within each group. However, between group comparisons revealed a statistically significant difference (*p* = 0.000), with the group receiving the highest dosage of tramadol (TT) exhibiting the deeper sedation with lowest sedation scores (RASS). These findings suggest a recovery characterized by the absence of excitatory phenomena. In this group, improved intraoperative analgesia and reduced postoperative stress may have contributed to facilitating earlier return to baseline behavior. This observation likely relates to the pharmacological mechanism of action of tramadol, which involves multiple pathways. Tramadol acts primarily as a weak µ-opioid receptor agonist and also inhibits the reuptake of serotonin and norepinephrine. At higher doses, the cumulative effect on these pathways may enhance central nervous system depression, leading to deeper sedation and the suppression of excitatory behaviors during recovery.

The dose-dependence of this effect can be attributed to the differential engagement of receptor systems at varying plasma concentrations. For instance, at lower doses, tramadol’s serotonergic and noradrenergic effects may predominate, potentially resulting in mild arousal or agitation. In contrast, at higher doses, its opioid activity becomes more pronounced, contributing to enhanced sedation and analgesia. This dose-related receptor engagement could explain why excitatory phenomena were absent only in the group receiving the highest tramadol dose. Regarding receptor types, tramadol primarily acts on µ-opioid receptors; agonism leads to analgesia and sedation [26].

Serotonin (5-HT) and norepinephrine (NE) transporter inhibition results in increased synaptic levels, contributing to analgesic effects but also potentially to excitatory responses if unbalanced. The observed reduction in excitatory phenomena may thus stem from a dominance of inhibitory mechanisms at higher tramadol concentrations. The enhanced intraoperative analgesia and reduced postoperative stress further support a smoother emergence from anesthesia, minimizing hyperactive behaviors.

Serotonin concentration trends provided further insights into the neurophysiological effects of the tested protocols. In the LL group (romifidine/ketamine/diazepam without tramadol), plasma serotonin levels significantly decreased 24 h post-surgery. This reduction could reflect a dysregulation or suppression of serotonergic activity associated with surgical stress or suboptimal recovery, consistent with findings in other species where decreased serotonin has been linked to reduced sleep quality and increased nociceptive sensitivity [27].

In contrast, the LT group (low-dose tramadol) showed a statistically significant increase in plasma serotonin levels postoperatively. This suggests that tramadol’s reuptake inhibition of serotonin may exert a measurable effect on systemic serotonin homeostasis [28]. This serotonergic modulation could underlie some of the observed clinical benefits, such as prolonged sedation and improved recovery [27].

Interestingly, in the TT group (high-dose tramadol), plasma serotonin levels remained stable, with no significant fluctuations observed over the 24 h period. This stability may indicate a plateau effect in serotonergic modulation or a homeostatic adjustment in response to higher levels of exogenous pharmacological stimulation [27]. Alternatively, it could reflect an optimal balance between serotonergic inhibition and opioid receptor activation, contributing to a more balanced recovery process.

Similar results were suggested in a study conducted on horses with osteoarthritis-related lameness treated with tapentadol as analgesic therapy. In the above study, plasma serotonin concentrations remained unchanged, comparable to those observed in the control group. Between-group comparisons of serotonin concentrations support these findings; statistically significant differences were found between the TT and LL groups, as well as between the LT and LL groups. The LL group consistently showed lower serotonin levels than the tramadol-treated groups, further highlighting the likely impact of tramadol on serotonergic activity and, potentially, on the modulation of the sleep–wake rhythm and the quality of recovery [23].

These findings are in line with previous research suggesting that serotonin plays a complex and biphasic role in the regulation of sleep and wakefulness [25]. While previous theories postulated serotonin as a sleep-promoting neurotransmitter, subsequent evidence has indicated that its effects are highly context-dependent, with both excitatory and synchronizing influences dependent on brain region, receptor subtype, and systemic neurochemical milieu [26].

The current results may be interpreted within this framework: tramadol’s action on serotonergic pathways may enhance the depth of sedation and postoperative recovery by promoting sleep-like states and modulating stress-related neurotransmission.

Taken together, the data suggest that tramadol, when integrated into a romifidine/ketamine/diazepam-based sedation protocol, contributes to improved sedation depth, more favorable postoperative recovery profiles, and modulation of serotonin levels in swine. This effect may reflect a broader impact on the neurochemical substrates of sleep and recovery, highlighting serotonin’s potential as a biomarker in the assessment of sedation and anesthesia. Surgical procedures induce inflammation and oxidative stress, and, in a recent study, serum concentrations of malondialdehyde (MDA), a marker of oxidative stress, were measured in dogs subjected to ovariectomy and were correlated with plasma concentrations of serotonin, which was considered a biomarker of inflammation and sedation quality [29]. The study revealed a strong positive linear relationship between MDA and serotonin levels, supporting the hypothesis that serotonin may be considered as a biomarker for pharmacologically induced sleep quality and, consequently, for the perception of nociceptive stimuli [30].

Although this study suggests that a serotonergic mechanism may underlie the observed benefits, it did not include direct electrophysiological recordings (e.g., EEG) or other objective measures capable of confirming the presence of sleep-like states or neural synchronization as the Bispectral Index (BIS).

BIS is an EEG-derived numerical scale ranging from 0 to 100, used to objectively assess the depth of anesthesia. Values between 40 and 60 are considered optimal for general anesthesia, indicating unconsciousness and amnesia, while lower values reflect deeper anesthetic states with increased risk of adverse effects. BIS monitoring aids in titrating anesthetic agents to avoid under- or over-dosage and is used alongside clinical signs to guide anesthetic management. The system employs a frontal adhesive sensor connected to a monitor that processes EEG data in real time. Limitations include susceptibility to artifacts and reduced reliability in pediatric or neurologically impaired patients. BIS should be considered a complementary tool rather than a substitute for clinical judgment [31]. This constitutes a notable limitation that should be addressed in future research through the application of advanced neurophysiological techniques. Nonetheless, the integration of behavioral, physiological, and biochemical markers offers compelling indirect evidence supporting a serotonin-mediated enhancement of sleep quality.

The study highlights several significant limitations that affect the interpretability and generalizability of the results. Firstly, ambient temperature may have influenced the observed outcomes. Given that this was a field-based clinical study, it was not feasible to replicate controlled experimental conditions. This introduces a notable confounding variable, as thermoregulation plays a critical role in pharmacokinetics and anesthetic response, particularly in swine [32].

Secondly, the limited availability of licensed anesthetic and analgesic agents for food-producing animals represents a regulatory constraint that impacts both experimental design and clinical veterinary practice. This shortage necessitates increased attention to the development of balanced, effective, and safe sedation and anesthesia protocols, constituting an important methodological limitation in this research field.

The protocol used in the present study is a general anesthesia protocol. It is plausible that the combination of an α2-agonist, a dissociative agent, and a benzodiazepine administered uniformly to all animals resulted in an anesthesia protocol sufficiently deep to blunt nociceptive responses. This may account for the absence of significant autonomic changes, such as increases >20% in heart rate, respiratory rate, or blood pressure, which are commonly used as surrogate markers of intraoperative nociception. We acknowledge this possibility and agree that the multimodal anesthetic regimen employed could have masked physiological indicators of nociceptive input, thereby influencing the interpretation of intraoperative responses. This point warrants further consideration in future studies aimed at isolating the specific contributions of each anesthetic component.

An additional limitation of the study lies in the use of subjective scales for assessing both depth of anesthesia and sedation. However, the inclusion of biomarker analysis provides support for more objective evaluation.

We recognize the limitations associated with intramuscular administration of diazepam, including variable absorbability, as reported in the literature [33]. However, our decision to use diazepam in this study was based on its extensive clinical use and well-characterized pharmacological profile. Intranasal administration of benzodiazepines in swine appears to be less stressful and may represent a valid alternative to intramuscular administration [34].

## 5. Conclusions

In conclusion, the addition of tramadol to a romifidine/ketamine/diazepam protocol enhances anesthesia quality and is likely associated with the modulation of serotonin levels, supporting its role in improving sleep-like states and postoperative recovery. From a clinical perspective, tramadol can be strategically incorporated into multimodal sedation protocols for swine undergoing field surgery such as hernia repair at doses calibrated to balance its sedative and analgesic effects without inducing excessive serotonergic stimulation. In this study, the higher dose of tramadol (4 mg/kg) resulted in a more prolonged depth of anesthesia and lower postoperative agitation, suggesting its suitability in procedures of moderate-to-high invasiveness. The lower dose (2 mg/kg) was associated with an increase in plasma serotonin concentration postoperatively, which may reflect a more active serotonergic engagement, contributing to enhanced recovery quality and potentially indicating a greater depth of anesthesia. Serotonin may serve as a promising biomarker in assessing the neurophysiological quality of sleep, offering a new avenue for refining anesthesia protocols in veterinary practice. We also believe that correlating scores related to the depth of anesthesia and postoperative sedation with serotonin concentrations could strengthen the hypothesis of serotonin’s role as a biomarker of quality sleep. These findings reinforce the potential of multimodal approaches in achieving balanced anesthesia with the best animal welfare outcomes.

## Figures and Tables

**Table 1:** Depth of anesthesia scores (data expressed with median and range): T0 baseline (awake subject); T1 after sternal recumbency; T2 after placement in dorsal recumbency; T3 at the time of skin incision; T4 during muscle incision; T5 at hernia sac opening and herniorrhaphy; T6 during muscle and subcutaneous tissue closure; and T7 at the time of skin suturing. * differences along the timeline (*p* < 0.05).

	Depth of Anesthesia Scores							
Group	T0	T1	T2	T3	T4	T5	T6	T7
LL	0 (0-0)	2 (2-2) *	3 (3-3) *	3 (3-3) *	3 (3-3) *	2 (2-2) *	2 (2-2) *	2 (2-2) *
LT	0 (0-0)	3 (3-3) *	3 (3-3) *	3 (3-3) *	3 (3-3) *	3 (3-3) *	3 (3-3) *	3 (3-3) *
TT	0 (0-0)	3 (3-3) *	3 (3-3) *	3 (3-3) *	3 (3-4) *	3 (3-3) *	3 (3-3) *	3 (3-3) *

**Table 2:** Post-operative sedation scores at the end of surgery and in post-operative timepoints (data expressed with median and range). T0 baseline (awake subject); T1 after sternal recumbency; T2 after to dorsal recumbency; T3 at the time of skin incision; T4 during muscle incision; T5 at hernia sac opening and herniorrhaphy; T6 during muscle and subcutaneous tissue closure; and T7 at the time of skin suturing.^α^ Differences between LL vs. TT; ^β^ differences between LT vs. TT (*p* < 0.05).

	Post-Operative Sedation		
Group	End of Surgery	2 h After Surgery	4 h After Surgery
LL	1 (1-1) ^α^	1 (1-1) ^α^	1 (1-1) ^α^
LT	−2 (−2/−2) ^β^	−1 (−1/−1) ^β^	−1 (−1/−1) ^β^
TT	−3 (−3/−3)	−2 (−2/−2)	−2 (−2/−2)

**Table 3:** Temporal and intergroup variations in 5-HT concentrations before and after surgery * Statistical differences along the timeline; ^α^ differences between LL vs. TT; ^β^ differences between LT vs. TT. Data are expressed as mean ± standard deviation. (*p* < 0.05).

	5-HT (ng/mL)	
Group	Baseline	24 h Post-Surgery
LL	36.00 ± 0.71 ^α^	35.28 ± 0.26 *^α^
LT	34.75 ± 1.19 ^β^	35.89 ± 1.05 *^β^
TT	36.56 ± 1.71	36.89 ± 0.13

## Data Availability

The data presented in this study are available on request from the corresponding author.
